# Protocol for an economic evaluation alongside the University Health Network Whiplash Intervention Trial: cost-effectiveness of education and activation, a rehabilitation program, and the legislated standard of care for acute whiplash injury in Ontario

**DOI:** 10.1186/1471-2458-11-594

**Published:** 2011-07-27

**Authors:** Gabrielle van der Velde, Pierre Côté, Ahmed M Bayoumi, J David Cassidy, Eleanor Boyle, Heather M Shearer, Maja Stupar, Craig Jacobs, Carlo Ammendolia, Simon Carette, Maurits van Tulder

**Affiliations:** 1 Toronto Health Economics and Technology Assessment (THETA) Collaborative, University of Toronto, Toronto, ON Canada; 2 Institute for Work & Health, Toronto, ON Canada; 3 Division of Health Care and Outcomes Research, Toronto Western Research Institute, University Health Network, Toronto, ON Canada; 4 Dalla Lana School of Public Health, University of Toronto, Toronto, ON Canada; 5 Centre for Research on Inner City Health, Keenan Research Centre in the Li Ka Shing Knowledge Institute and Division of General Internal Medicine, St. Michael's Hospital, Toronto, ON Canada; 6 Department of Health Policy Management and Evaluation, University of Toronto, Toronto, ON Canada; 7 Department of Medicine, University of Toronto, Toronto, ON Canada; 8 Department of Medicine, Mount Sinai Hospital, Toronto, ON Canada; 9 Division of Rheumatology, University Health Network, Toronto, ON Canada; 10 Department of Health Sciences, Faculty of Earth & Life Sciences, VU University, Amsterdam, Netherlands

**Keywords:** budget impact analysis, cost-effectiveness analysis, expected value of perfect information, quality-adjusted life year, whiplash-associated disorders, whiplash injury, treatment

## Abstract

**Background:**

Whiplash injury affects 83% of persons in a traffic collision and leads to whiplash-associated disorders (WAD). A major challenge facing health care decision makers is identifying cost-effective interventions due to lack of economic evidence. Our objective is to compare the cost-effectiveness of: 1) physician-based education and activation, 2) a rehabilitation program developed by Aviva Canada (a group of property and casualty insurance providers), and 3) the legislated standard of care in the Canadian province of Ontario: the Pre-approved Framework Guideline for Whiplash developed by the Financial Services Commission of Ontario.

**Methods/Design:**

The economic evaluation will use participant-level data from the University Health Network Whiplash Intervention Trial and will be conducted from the societal perspective over the trial's one-year follow-up. Resource use (costs) will include all health care goods and services, and benefits provided during the trial's 1-year follow-up. The primary health effect will be the quality-adjusted life year. We will identify the most cost-effective intervention using the incremental cost-effectiveness ratio and incremental net-benefit. Confidence ellipses and cost-effectiveness acceptability curves will represent uncertainty around these statistics, respectively. A budget impact analysis will assess the total annual impact of replacing the current legislated standard of care with each of the other interventions. An expected value of perfect information will determine the maximum research expenditure Canadian society should be willing to pay for, and inform priority setting in, research of WAD management.

**Discussion:**

Results will provide health care decision makers with much needed economic evidence on common interventions for acute whiplash management.

**Trial Registration:**

http://ClinicalTrials.gov identifier NCT00546806 [Trial registry date: October 18, 2007; Date first patient was randomized: February 27, 2008]

## Background

Whiplash injury affects 83% of persons involved in a traffic collision [1]. In the short-term, this injury leads to whiplash-associated disorder (WAD), a clinical syndrome characterized by neck pain and clusters of physical and psychological symptoms [2,3]. In the long-term, whiplash injury increases the incidence of future health problems. Persons with a history of whiplash are more likely to suffer from future neck pain, headaches, low back pain, shoulder pain and sleep disturbances, compared to those without a history of whiplash [1,4-6]. WAD represents an important and growing burden both in terms of direct medical costs (associated with health care use) and indirect costs (associated with productivity changes, lost earnings capacity, and time contributed by caregivers) [7]. Most industrialized countries have seen a rise in the incidence of hospital visits for traffic-related WAD over the past 20 years [8]. In the Netherlands, the average annual incidence has increased 10 times over a twenty-year period, from 3.4 visits per 100,000 inhabitants (1970-1974) to 40.2 visits per 100,000 (1990-1994) [9]. In Sweden, the annual cumulative incidence of emergency visits for WAD has increased from 83 per 100,000 inhabitants (1985-1986) to 302 per 100,000 (1997-1998) [8].

In the Canadian province of Ontario, health care resources used to manage traffic injuries within the automobile insurance system exceed those used by the Workers Safety and Insurance Board system for similar injuries [10]. A recent assessment of sprain and strain (mainly whiplash) injuries from 2004 - 2007 by the Ontario insurance industry found that despite a 20% increase in costs over the 3-year time period, the percent of claims closed within the expected time frame for soft tissue injuries declined dramatically [10]. Thus WAD claims have increased in duration, despite the fact that more health care resources have been directed to WAD management. Drivers in Ontario consequently pay much higher automobile insurance premiums compared to drivers in other Canadian provinces where automobile insurance is also sold by private companie [11]. About, 5% of Ontario motorists' disposable income is spent on auto insurance, whereas motorists in Alberta and the Atlantic provinces pay 3% [10]. Recent scientific studies have also observed that increasing health care use has not led to improved health outcomes in WAD patients. Two population-based studies in Saskatchewan found that higher health care use was associated with substantial delays in recovery that were not explained by injury severity or other factors [12,13]. Another study found that when compared to usual care, specialized rehabilitation programs did not benefit recovery from WAD [14]. A 2006 randomized trial compared education and advice by general practitioners (mean number of visits = 3.0) to education and exercises by physiotherapists (mean number of visits = 12.7) in patients with WAD lasting more than four weeks [15]. Patients in the general practitioner group reported lower levels of neck pain and headache intensity than patients treated by physiotherapists [15].

Thus, a major challenge facing health care decision makers is identifying cost-effective interventions for WAD. But there are no economic evaluations of WAD interventions in the published literature. The recently published systematic review of the neck pain literature by the Bone and Joint Decade 2000-2010 Task Force on Neck Pain and Its Associated Disorders did not identify a single cost-effectiveness analysis of WAD treatments [16]. Our objective is to conduct an economic evaluation alongside a pragmatic randomized trial that evaluates physician-based education and activation and two rehabilitation programs for managing WAD in real-world conditions.

## Methods/Design

This paper describes the protocol of an economic evaluation alongside the University Health Network

Whiplash Intervention Trial (UHN WIT). A complete description of the UHN WIT protocol has

been published elsewher [17]. and is summarized below.

### University Health Network Whiplash Intervention Trial Protocol

The UHN WIT is a three-arm randomized trial designed to compare the effectiveness of education and activation by a physician and two rehabilitation programs of care in patients with recent Grade I or II WAD (Table 1). Selection criteria are listed in Table 2 and the interventions are summarized below [17]. The UHN WIT protocol was approved by the University Health Network Research Ethics Board (January 4, 2008).

**Table 1:** The Québec Classification of Whiplash-associated Disorder [2].

Grade	Clinical Presentation
0	No neck symptoms, no physical sign(s)
I	Neck pain, stiffness or tenderness only, no physical sign(s)
II	Neck symptoms and musculoskeletal sign(s)*
III	Neck symptoms and neurologic sign(s)†
IV	Neck symptoms and fracture or dislocation

*Musculoskeletal signs include decreased range of motion and point tenderness.

†Neurologic signs include decreased or absence of deep tendon reflexes, weakness, and sensory deficits.

Symptoms and disorders that can be manifested in all grades include deafness, dizziness, tinnitus, headache, memory loss, dysphagia, and temporomandibular pain.

**Table 2:** Selection criteria for the University Health Network Whiplash Intervention Trial [17].

Inclusion criteria
18 years of age or older. Reside or work in the Greater Toronto Area, Mississauga, Burlington, Cambridge, Kitchener, Ajax, Pickering, or the Durham region in the province of Ontario, Canada. Make an insurance claim for physical injury to Aviva Canada* between February 1, 2008 to March 31, 2012 within 21 days of the traffic collision.
Report an average neck pain since the accident of at least 3 on a 0-10 numerical rating scale.
Are injured in a motor vehicle collision with clinician-assessed Grade I or II WAD. Are able to provide written informed consent and complete interviews in English (translators will be available to assist the participant if the claimant experiences difficulty understanding specific items on the questionnaire).

**Exclusion criteria**

Fracture/dislocation of the spine or any major bone.
Head trauma associated with loss of consciousness.
Past whiplash or work-related neck injury within the year prior to current WAD injury.
Active systemic diseases (cancer, inflammatory arthritis, disorders of central nervous system).
Previous neck surgery.
Received treatment from a physiotherapist or chiropractor for neck pain in the three months preceding the motor vehicle collision.
Does not reside or work in Greater Toronto Area, Mississauga, Burlington, Cambridge, Kitchener, Ajax, Pickering, or the Durham region in the province of Ontario, Canada.

WAD = Whiplash-associated Disorder (See Table 1 for definition of Whiplash-associated Disorder grading system).

*Aviva Canada is a group of property and casualty insurance providers.

#### Physician-based Education and Activation

The Education and Activation intervention is designed to promote self-efficacy and early return to normal activities of daily living. It reflects current guidelines and recommendations for the management of WAD [18,19]. A primary care physician provides reassurance, education about WAD's favorable prognosis, encouragement to resume activities of daily living, and a recommendation to perform home stretching exercises. The physician prescribes medicinal or non-medicinal pain relief modalities (heat/ice), a physiotherapy visit for further instruction on home exercises, or both, based on clinical judgment. The physician determines whether the participant can be discharged or a follow-up visit is required at the end of the first visit. The participant is advised to contact the physician if the complaint persists or worsens.

If follow-up visits are required, the participant is re-assessed by the same physician (if the physician is unavailable, another physician associated with the trial re-assesses the participant). The intervention above is repeated and adapted to the participant's status according to the physician's clinical judgment (e.g., alternate pain medication is prescribed). Participants who do not recover six weeks post-collision, or improve but require more care, are referred for a multidisciplinary evaluation and enter the interdisciplinary rehabilitation intervention stream (described in the Soft Tissue Injury Care Model section below).

#### Pre-approved Framework Guideline for Grade I and II Whiplash-associated Disorders

The Pre-approved Framework is a clinical management guideline developed by the Financial Services Commission of Ontario, an arm's length agency of the Ministry of Finance which regulates traffic insurance in the province of Ontario. It is the legislated standard of care for Grade I and II WAD in Ontario. It focuses on the provision of interventions to manage pain and disability through functional restoration [20]. The intervention is administered by a physiotherapist and includes: 1) reassurance, 2) education, 3) home exercises, and 4) encouragement to resume normal activities of daily living. The physiotherapist may also provide: 1) exercise and functional activities, 2) mobilization and manipulation, 3) non-medicinal pain management modalities (heat/ice, massage therapy), and 4) coping skills education. The frequency and number of visits is based on the physiotherapist's clinical judgment, but does not exceed 10 visits within the first three weeks and 9 visits three to six weeks after the collision date. The limit on the number of visits is a restriction of the Pre-approved Framework, not of the trial protocol.

Participants with clinically important functional limitations are eligible for a functional assessment of their home, work, or school environment. Based on the assessment, an occupational therapist develops an intervention that may include: 1) recommendation to use aids or devices, 2) minor modifications at the work, home, or school environment, 3) instructions on adaptive strategies or alternate approaches to fulfill functional tasks, and 4) specific functional activities to increase tolerance. Participants who report clinically significant improvement in the first six weeks of care, but have not recovered, can receive up to four additional treatments over a two-week period. Participants who have not recovered in the first six weeks of care are re-evaluated and a new plan of management is developed by the physiotherapist.

#### Soft Tissue Injury Care Model

The Soft Tissue Injury Care Model is a staged multidisciplinary rehabilitation program developed by Aviva Canada (a group of property and casualty insurance providers) as an alternative to the Pre-approved Framework guideline. A physiotherapist leads the intervention which includes three integrated and sequentially-administered rehabilitation phases: 1) acute-sub acute care, 2) multidisciplinary evaluation, and 3) interdisciplinary rehabilitation. During the acute-sub acute phase (first six weeks post-collision), care is delivered by physiotherapists, and if necessary, kinesiologists and massage therapists. Care consists of: 1) reassurance, 2) education, 3) home exercises, 4) physiotherapy modalities, and 5) massage therapy. A maximum of 9 treatments (including massage therapy) during the first three weeks post-collision and an additional 8 sessions (including massage therapy) between the third and sixth week post-collision is provided. The limit on the number of visits is a restriction of the Soft Tissue Injury Care Model, not of the trial protocol. The type and frequency of treatment prescribed by the physiotherapist is based on his/her assessment. During this phase, the physiotherapist may recommend an in-home or job-site functional assessment.

Patients who require treatment beyond the acute-sub acute phase are referred for a multidisciplinary evaluation. The purpose of the multidisciplinary evaluation is to identify barriers to recovery and recommend the appropriate treatment through an interdisciplinary rehabilitation program. The interdisciplinary rehabilitation program has three specific goals: 1) overcome psychosocial barriers to return to function, 2) physical restoration, and 3) functional restoration. The program includes up to five weeks of daily intervention (each session may last up to 51/2 hours). Frequency, duration and type of care are determined by the interdisciplinary team and may include a job-site assessment. Services provided during interdisciplinary rehabilitation may include: 1) education, 2) reassurance, 3) goal setting and advice on self-management, 4) psychological counseling and stress management, 5) relaxation therapy, psychotherapy and family counseling, 6) cognitive behavioral therapy, 7) instruction on pain management techniques, and 8) strength, endurance, flexibility or cardiovascular exercises.

### Quality Control

All physicians and physiotherapists are trained by a trial coordinator during a formal training session. The session consists of a description of the study and instruction on the standardized intervention protocols. Clinicians receive printed materials to refer to. A trial coordinator conducts intermittent file audits and protocol reviews with clinicians to ensure adherence with the protocol.

### Data Collection, Follow-up, and Outcomes

Table 3 summarizes data collected during the trial. Participants are assessed at 6 weeks, and 3, 6, 9 and 12 months from baseline. The UHN WIT primary outcome is time-to-recovery measured by a global self-perceived recovery question - a reliable, valid, and responsive measure of health status in patients with musculoskeletal disorders [21,22]. Participants are asked: "How well do you feel you are recovering from your injuries?" at each follow-up. They are requested to select one of seven choices: 1) completely better; 2) much improved, 3) slightly improved, 4) no change, 5) slightly worse, 6) much worse, and 7) worse than ever. Participants who respond to have "completely recovered" or are "much improved" are considered to have recovered. Time-to-recovery is measured as number of days between the date of injury and the first follow-up date that a participant reports to be "completely recovered" or "much improved".

**Table 3:** Outcomes and variables measured at baseline and follow-up interviews.

Measures	Baseline	6 Weeks	3 Months	6 Months	9 Months	12 Months
Socio-demographic characteristics	x					
Accident information	x					
Past history of neck pain and whiplash	x					
Health care after accident	x					
Co-morbidity questionnaire	x					
Neck pain intensity	x	x	x	x	x	X
Whiplash disability questionnaire	x	x	x	x	x	X
SF-36 (acute, v2)	x	x	x	x	x	x
CES - Depression scale	x	x	x	x	x	x
Health state preference (rating scale)	x	x	x	x	x	x
Expectation of recovery	x	x	x	x	x	x
Global self-perceived recovery question	x	x	x	x	x	x
Work status	x	x	x	x	x	x
Lawyer or paralegal involvement	x	x	x	x	x	x
Satisfaction with care		x	x	x	x	x
Co-interventions		x	x	x	x	x

CES = Center of Epidemiological Studies; SF-36 (acute, v2) = Medical Outcomes Study 36-item Short-Form health survey (acute, v2)

Secondary outcomes include the following. A recurrence occurs when a participant who reports to have recovered subsequently reports that s/he is: 1) slightly worse, 2) much worse, or 3) worse than ever. Neck pain intensity is measured with an 11-point numerical rating scale [23]. The numerical rating scale is a measure of pain intensity anchored by two extremes of pain intensity varying from 0 (no pain) to 10 (pain as bad as it could be). Disability is measured with the Whiplash Disability Questionnaire [24]. The questionnaire includes 13 Likert scales scored from 0 to 10 with higher global scores indicating more disability. The acute 36-item Medical Outcomes Study Short-Form Health Survey (version 2) is used to measure health-related quality-of-life [25-27]. Depressive symptoms are measured with the Center for Epidemiological Studies-Depression Scale [3,28,29]. The scale is scored from 0 to 60 with higher scores indicating greater depressive symptomatology [30,31]. Co-interventions are measured by asking participants to self-report the type of health care provider consulted outside of the trial and the frequency of these visits. Participants are also asked to self-report medication use, including over-the-counter medication and drugs prescribed by heath providers not associated with the trial. A cross-validation will be conducted by checking self-reported co-interventions against the out-of-pocket expenses submitted by participants to Aviva Canada for reimbursement. (See 'Resource Use (Costs)' section below for more information on out-of-pocket expenses paid by the automobile insurer).

#### Statistical Analysis of Trial Effectiveness Data

The primary analysis will be conducted according to the intention-to-treat principle. It will compare time-to-recovery across intervention arms using the Kaplan-Meier method, reporting median time-to-recovery and 95% confidence intervals [32]. The mixed-effect Cox proportional hazards model will be used to measure the relative effectiveness of Education and Activation, Pre-approved Framework, and Soft Tissue Injury Care Model [33-36]. If participant baseline characteristics vary between interventions, a multivariable mixed-effect Cox proportional hazards model will be used to control for differences between groups. The proportional hazards assumption of a constant hazard ratio will be tested using graphical approaches, goodness-of-fit tests, and time-dependent variables. A stratified Cox procedure will be used if the assumption does not hold [37].

Recurrences will be reported as rates (number of participants that report a recurrence per 28 days). Rates will be used because differences in participants' time-to-recovery could create a bias that favors less effective interventions (those with longer average time-to-recovery) since, in participants randomized to these interventions, there will be less observation time to observe potential recurrences. Time-to-recurrences will also be compared in a Cox proportional hazards model as above, with an additional covariate to account for differences in average time-to-recovery across interventions.

### Economic Evaluation alongside the UHN WIT

The primary objective is to compare the relative cost-effectiveness of: 1) the legislated standard of care in Ontario for acute WAD, 2) physician-based Education and Activation, and 3) the Soft Tissue Injury Care Program, for persons with recent WAD injury, using participant-level data from the UHN WIT and Aviva Canada administrative claim files. Secondary objectives including conducting sensitivity analyses, an expected value of perfect information analysis, and a budget impact analysis. A societal perspective will be adopte [38,39]. The time horizon will be 12 months (the maximum follow-up for the trial).

### Resource Use (Costs)

Ontario automobile insurance regulations specify that extended health care benefits (e.g., benefits provided by employers on top of public health care benefits) are used first when paying for traffic injury treatment. When extended benefits are exhausted, automobile benefits are used. However in this trial, Aviva Canada pays for all health care, including those generally paid by extended care benefits.

Relevant costs will include all health care goods and services, and benefits provided by Aviva Canada during the study's 12-month follow-up period (Table 4). Participants will not be requested to complete cost diaries since patient out-of-pocket expenses are paid by automobile insurance providers in Ontario. Therefore, expenses submitted by participants to Aviva Canada will be used as a proxy to a cost diary. At all follow-ups, participants also complete a questionnaire about access to health care for their whiplash injury beyond health care provided by the trial (i.e., publicly or privately funded or unfunded health provider services). Costs will be assigned to health provider visits using: 1) Ontario Ministry of Health and Long-term Care Schedule of Benefits (for physician visits, which are publicly funded) or 2) regulated health professions' recommended fee schedules (for acupuncture, chiropractic, massage therapy, naturopathy, physiotherapy visits, etc., which are not publicly funded). Unit values for over-the-counter and prescription drugs will be obtained from a wholesale distributor catalogue.

**Table 4:** Resource use (costs) to be collected.

Costs	Examples
	
Health care provider fees	Initial assessment, follow-up visits.
	
Fees for completing standardized automobile insurance claims forms	Treatment Plan (OCF-18), Discharge and Status Report (OCF-24).
	
Health services (block fees)	In-home assessment, functional evaluation, job site assessment, home exercise prescription, multidisciplinary evaluation, workplace/home modification assessment, home exercise prescription, multidisciplinary evaluation, workplace/home/vehicle modification assessment, return to work support, education programs, life skills and job retraining, counseling and coaching, stress management, relaxation therapy, family counseling, cognitive behavioral therapy, pain management.
	
Patient out-of-pocket expenses	Over-the-counter medications, prescription drugs, portable electrotherapy devices, orthotics, assistive devices, health care supplies, treatment-related expenses.
	
Diagnostic imaging	Radiography, magnetic resonance imaging, ultrasound.
	
Benefits	Non-earner, dependent care, caregiver, housekeeping and home maintenance, income replacement, attendant care, lost educational expenses, expense of visitors, long-term care, excess economic loss.
	
Independent health examination	Chiropractic, medical, occupational therapy, physical therapy, psychological.
	

OCF = Ontario Claim Form

Cost data will be extracted from participants' insurance claim file by a blinded research associate unaffiliated with Aviva Canada using a standard data extraction form. The research associate will pilot data extraction by extracting data twice from the files of the first 50 participants to test intra-rater reliability. A value of at least 0.8 will be considered adequate for the quadratic weighted Kappa statistic (in the case of categorical data) and intra-class correlation coefficient (in the case of continuous data). Data extraction will be modified if problems are identified. Cost data will be linked with trial data using the unique study identification number assigned to each participant at enrollment. Costs will be reported in Canadian dollars and standardized for inflation to the most recent available rates after the study has ended, using the Bank of Canada Consumer Price Index and adjusted for censoring using the Kaplan-Meier sample average estimator [40-42].

### Health Effects (Outcomes)

We will use the quality-adjusted life year (QALY) as the primary health outcome for our analysis. SF-6D scores - derived from SF-36 response data - will be used to estimate participants' preference-based quality-of-life (utilities) over the 12-month follow-up period [43]. These scores will be converted into a within-trial estimate of participant-level QALYs using the area-under-the-curve approach, with linear interpolation between assessment points and adjusting for potential differences in baseline characteristics [44]. The number of unrecovered days will be derived from the trial's primary outcome 'time-to-recovery' based on participants' self-perceived recovery. Mean number of unrecovered days will be adjusted for censoring using the Kaplan-Meier product limit estimator [32,37]. The number of recurrences will be derived from the trial's secondary outcome, standardized to a rate (number of recurrences per 28 days) to account for differences in participants' time-to-recovery.

### Statistical Analysis

Participant characteristics and health resources consumed will be reported by intervention group, using descriptive statistics (measures of central tendency and variance).

#### Primary Cost-effectiveness Analysis

Costs and health effects will not be discounted for the primary analysis since the time horizon is 12-months. Following current recommendations [38]. costs related to productivity changes (e.g., income replacement, excess economic loss benefits) will not be included in the primary cost-effectiveness analysis. Productivity changes will be reported separately, including quantities (e.g., days of work lost) and prices used to value the quantities. We will calculate mean cost per patient and mean effect per patient by intervention group, based on initial intervention assignment, with costs and effects adjusted for censoring [41]. We will identify the cost-effectiveness of the interventions using the incremental cost-effectiveness ratio and incremental net-benefit. Confidence ellipses (50%, 75%, 95%) and cost-effectiveness acceptability curves will represent the uncertainty around these statistics, respectively.

Incremental cost-effectiveness will be computed as the ratio of the difference in mean costs (incremental cost, ΔC) to the difference in mean health effects (incremental effect, ΔE), and will provide incremental cost-effectiveness ratios (ICERs):

ICERs will represent the additional cost per additional QALY gained, additional cost per additional unrecovered day averted, and additional cost per additional recurrence over a 28 day period of one intervention compared to another, from least costly to most costly. ICERs will be plotted on a cost-effectiveness plan [45,46]. with uncertainty represented by confidence ellipses [47-49]. which are a two-dimensional generalization of the confidence interval. We will estimate the distribution of mean incremental costs and effects by the non-parametric re-sampling technique of bootstrapping with replacement from 10,000 replicates and presented as a scatter plot on the cost-effectiveness plane [48,49].

Whether the ICER is considered cost-effective by a decision maker depends on the maximum the decision maker is willing to pay for an extra unit of health effect (the threshold value, λ). An intervention that is cost-effective is thus defined as ICER < λ. The Incremental Net Benefit (INB) equatio [50]. computes the net benefit of a health effect in dollars, by valuing the additional health effect (ΔE) in dollars, and subtracting the associated additional cost (ΔC) as follows:

When the INB is positive, the value of a new treatment's extra benefits (ΔE × λ) outweighs its extra costs (ΔC), implying that the decision maker values the extra effect more than the extra cost (i.e., ΔE × λ > ΔC). Conversely, when INB is negative, the decision maker does not consider the extra benefit to be worth the extra cost.

We will use the net-benefit approac [50,51]. to measure the incremental cost-effectiveness of the interventions against a threshold value λ, where λ will be described as society's maximum willingness-to-pay for an additional: 1) QALY gained, 2) unrecovered day averted, and 3) recurrence averted over a 28 day period. We will compute INB over a range of willingness-to-pay threshold values based on $50,000 per QALY and other values cited in the health economics literature. Uncertainty will be represented by cost-effectiveness acceptability curves [47,52,53]. Cost-effectiveness acceptability curves are derived from the joint density of incremental costs and incremental effects for an intervention of interest and represent the proportion of the density where an intervention is cost-effective for a range of λ values [54]. In this study, the joint density will be obtained by non-parametric bootstrapping from the distribution of observed cost-effect pairs. Cost-effectiveness acceptability curves will show the probability that the interventions are cost-effective over a full range of values for λ for an additional unit of health effect.

We will examine baseline characteristics across intervention groups to determine whether randomization was successful in balancing observed baseline covariates. If there is unbalanced allocation in baseline characteristics, we will use net-benefit regression [55] which allows cost-effectiveness to be estimated directly in a regression framework. Net-benefit regression uses net-benefit as the dependent variable calculated from person-level effect (E_i_) and cost (C_i_) data for each patient i:

In its simplest form, regression is then used to estimate:

where α is the intercept term, t is an intervention dummy term (it equals 1 if the person received, for example, the new intervention and 0 for the usual care), and ε is the stochastic error term. The regression coefficient β provides the estimate of the standard incremental net-benefit statistic. As there are two new interventions to test, we will use two treatment dummies, t1 and t2 (with corresponding coefficients β_1 _and β_2_). The regression estimate of β_1 _will equal the incremental net benefit of physician-based Education and Activation compared to the Ontario legislated standard of care, and the regression estimate of β2 will equal the incremental net benefit of the Soft Tissue Injury Care Program compared to the Ontario legislated standard of care. Additional covariates can be added to adjust for unbalanced allocation in observed baseline covariates that could confound results [55]. Subgroups for whom new interventions are especially cost-effective can be identified through interaction terms (e.g., X*t).

#### Sensitivity Analyses

Sensitivity analyses will test the robustness of results to selected issues and assumptions. First, cost-effectiveness will be re-evaluated with costs related to productivity changes included as these costs will not be included in the primary analysis. Second, the sensitivity of the results to using SF-6D versus rating scale quality-of-life weights to calculate QALYs will be tested. The rating scale approach consists of asking participants to choose a number that best represents their current health state on a scale anchored from 0 (worst imaginable health state) to 100 (best imaginable health state) [56]. Finally, we will test assumptions about the long-term costs and effect of the interventions extrapolated beyond the 12-month time horizon, including the assumptions that: 1) after the 12-month period, the rate of recovery is identical, and 2) the interventions continue to confer the same rate of recovery. These assumptions will be extrapolated over a 5-year time horizon [57].

### Missing Data and Loss to Follow-up

Observations with incomplete baseline covariates will be deleted from the analyses. Participants with missing data on the primary outcome (self-perceived recovery) and preference-based quality-of-life (SF-6D) will have the result of their last observation carried forward. Sensitivity analyses will be conducted, where reasonably likely scenarios are considered (e.g., missing observations are assumed to have a distribution of outcomes including recovery, recurrence, and no recovery). Cost and effect data will be analyzed with two approaches: 1) only participants with complete data are included (complete case analysis), and 2) all study participants are included, where imputed values for costs and effects in participants with missing data will be used (multiple imputation) [58]. Results of the multiple imputation analysis will be compared to the complete case analysis [59,60].

### Expected Value of Perfect Information

Expected value of perfect information (EVPI) represents the maximum amount that a health care decision maker should be willing to pay for additional evidence to inform future decisions [38]. It focuses on the value of obtaining further information to reduce uncertainty. We will estimate EVPI per participant, multiply this value by the average annual number of WAD claims in Ontario over a previous five year period, and plot this resultant population EVPI for a range of willingness-to-pay thresholds. The results will provide the maximum expenditure that society or a health care decision maker should be willing to pay for, and inform priority setting in, WAD management research.

### Budget Impact Analysis

A budget impact analysis will be conducted to assess the cost of replacing the Pre-approved Framework Guideline (the current legislated standard of care for acute Grade I and II WAD in Ontario) with the physician-based Education and Activation and Aviva Canada's Soft Tissue Injury Care Model [61,62]. The total annual impact on the budget of the automobile insurer and subsequent implication to automobile premiums in Ontario will be considered using published underwriting ratios (an underwriting - or combined - ratio is a measure of an insurance company's profitability). Analytic assumptions will be based on published epidemiological WAD incidence rates, automobile insurance traffic injury rates, and data collected by this economic evaluation [1,10].

## Conclusion

This paper allows for the peer-review of the proposed methods and provides a transparent statement of the planned analyses. Results of this economic evaluation are expected to provide health care decision makers with necessary economic evidence on common interventions for acute whiplash.

## List of abbreviations

EVPI: expected value of perfect information; ICER: incremental cost-effectiveness ratio; INB: incremental net-benefit; QALY: quality-adjusted life year; UHN WIT: University Health Network Whiplash Intervention Trial; WAD: whiplash-associated disorders.

## Competing interests

The authors declare that they have no competing interests.

## Authors' contributions

AMB, EB, GvdV, HMS, JDC, MS, and PC participated in the conception and design of the study. The analysis was designed by AMB, EB, GvdV, JDC, and PC. All authors participated in drafting this manuscript and approved its final version.

## Pre-publication history

The pre-publication history for this paper can be accessed here:

http://www.biomedcentral.com/1471-2458/11/594/prepub
